# Clinical features and genetic analysis of a case series of skeletal ciliopathies in a prenatal setting

**DOI:** 10.1186/s12920-023-01753-y

**Published:** 2023-12-07

**Authors:** Ying Peng, Lin Zhou, Jing Chen, Xiaoliang Huang, Jialun Pang, Jing Liu, Wanglan Tang, Shuting Yang, Changbiao Liang, Wanqin Xie

**Affiliations:** Prenatal Diagnosis Center, National Health Commission Key Laboratory of Birth Defects for Research and Prevention, Hunan Provincial Maternal and Child Health Care Hospital, Changsha, 410008 Hunan China No. 53 Xiangchun Road,

**Keywords:** Ciliopathy, Short-rib polydactyly syndromes, *DYNC2H1*, *IFT172*, *WDR19*, Exome sequencing, Prenatal diagnosis

## Abstract

**Background:**

Short-rib polydactyly syndrome (SRPS) refers to a group of lethal skeletal dysplasias that can be difficult to differentiate between subtypes or from other non-lethal skeletal dysplasias such as Ellis-van Creveld syndrome and Jeune syndrome in a prenatal setting. We report the ultrasound and genetic findings of four unrelated fetuses with skeletal dysplasias.

**Methods:**

Systemic prenatal ultrasound examination was performed in the second or third trimester. Genetic tests including GTG-banding, single nucleotide polymorphism (SNP) array and exome sequencing were performed with amniocytes or aborted fetal tissues.

**Results:**

The major and common ultrasound anomalies for the four unrelated fetuses included short long bones of the limbs and narrow thorax. No chromosomal abnormalities and pathogenic copy number variations were detected. Exome sequencing revealed three novel variants in the *DYNC2H1* gene, namely NM_001080463.2:c.6809G > A p.(Arg2270Gln), NM_001080463.2:3133C > T p.(Gln1045Ter), and NM_001080463.2:c.337C > T p.(Arg113Trp); one novel variant in the *IFT172* gene, NM_015662.3:4540-5 T > A; and one novel variant in the *WDR19* gene, NM_025132.4:c.2596G > C p.(Gly866Arg). The genotypes of *DYNC2H1, IFT172* and *WDR19* and the phenotypes of the fetuses give hints for the diagnosis of short-rib thoracic dysplasia (SRTD) with or without polydactyly 3, 10, and 5, respectively.

**Conclusion:**

Our findings expand the mutation spectrum of *DYNC2H1, IFT172* and *WDR19* associated with skeletal ciliopathies, and provide useful information for prenatal diagnosis and genetic counseling on rare skeletal disorders.

**Supplementary Information:**

The online version contains supplementary material available at 10.1186/s12920-023-01753-y.

## Introduction

Short-rib polydactyly syndrome (SRPS) refers to a group of autosomal recessive skeletal dysplasias characterized by markedly short ribs with thoracic hypoplasia, short limbs, and variable presentation of polydactyly and metaphyseal and visceral anomalies. Histopathologically, SRPS, asphyxiating thoracic dysplasia (ATD; also known as Jeune syndrome) and Ellis van Creveld syndrome (EVC) belong to ciliopathies with major skeletal involvement [1]. These disorders exhibit phenotypic overlap, presenting a challenge for clinical diagnosis, especially in the setting of prenatal diagnosis [2, 3].

SRPS, ATD, EVC and Mainzer-Saldino syndrome (MZSDS) are also summarized under the term short-rib thoracic dysplasia (SRTD) with or without polydactyly [4–6]. The phenotype-genotype relationships of SRTD types 1–23 can be found in the Online Mendelian Inheritance in Man (OMIM) database (Supplementary Material Table S1).

Cilia are the fine protrusive structures of cell surface that play important roles in development and function of many organs including bones [7]. The movement of substances within cilia, known as intraflagellar transport (IFT), is critical for the assembly and maintenance of cellular structures. The IFT particles responsible for bi-directional transportation within cilia are composed of complex A (IFT-A) and complex B (IFT-B) [8]. Among the ciliary proteins associated with SRTD, WDR35, IFT140, WDR19, IFT43, and TTC21B are components of the complex A; IFT80, IFT172, IFT52, and IFT81 take participation in the complex B; DYNC2H1, WDR60, WDR34, DYNC2LI1, and TCTEX1D2 are IFT-dynein motor proteins; NEK1, CEP120, and KIAA0586 are located at the basal body of primary cilia; and INTU is a ciliogenesis and planar polarity effector (CPLANE) protein [9]. Hence, the genetic architecture of skeletal ciliopathies involves both the structural and regulatory proteins of cilia.

The present study reports four prenatal cases of SRPS/SRTD as implicated by ultrasound findings, which provide useful information regarding the novel variants of ciliopathies-associated genes *DYNC2H1*, *IFT172*, and *WDR19*.

## Materials and methods

### Study participants

Four pregnant women with fetal ultrasound abnormalities including thoracic dysplasia, short ribs, and short long bones with or without organ anomalies were included in the present study.

### SNP array for CNV detection

Preparation of genomic DNA from amniocytes or fetal tissue was performed using QIAamp DNA Blood Mini Kit or QIAamp DNA Mini Kit (QIAGEN, Germany). The purified DNA was then processed using CytoScan 750 K reagent kit and subsequently loaded onto the CytoScan 750 K array for hybridization following the manufacturer’s instructions (Applied Biosystems, Thermo Fisher Scientific). The CytoScan 750 K array was preloaded with 200,000 SNPs and 550,000 non-polymorphic probes for copy number analysis. The Chromosome Analysis Suite (ChAS) software (available at https://www.thermofisher.com/) was used to analyze the raw data. CNVs ≥ 100 kb were chosen for blast analysis and annotated with the reference databases including DGV (http://dgv.tcag.ca/), DECIPHER (https://decipher.sanger.ac.uk/), OMIM, UCSC (https://genome.ucsc.edu/hg19), ClinVar and PubMed.

### Exome sequencing

Novaseq6000 platform (Illumina, San Diego, USA) with 150 bp pair-end reads was used for sequencing the genomic DNA from the fetus and parents. Qualified genomic DNA was sheared into around 150 bp fragments, then blunt-ended and added with deoxyadenosine (dA) to the 5’ tails, followed by adding adaptors to the ends of DNA double strands. The library was amplified by polymerase chain reaction (PCR) and then hybridized with a pool of biotin-labeled oligo probes specific for exons. DNA-probes hybrids were captured using streptavidin magnetic beads and a round of PCR was used to amplify the library to sufficient levels for sequencing. Raw image files were processed using CASAVA v1.82 for base calling and generating raw data with sufficient CCDS coverage (95.87%–98.88%, for depth ≥ 20). The sequencing reads were aligned to the human reference genome (hg19/GRCh37) using Burrows–Wheeler Aligner tool and PCR duplicates were removed by using Picard v1.57 (http://picard.sourceforge.net/). The interpretation of sequence variants were conducted, referring to the American College of Medical Genetics and Genomics (ACMG) guidelines [10, 11] and the Enliven® Variants Annotation Interpretation System authorized by Berry Genomics (Beijing, China). Variants with a frequency > 1% in the databases including 1000 Genomes (http://browser.1000genomes.org), Exome Aggregation Consortium (ExAC) and Genome Aggregation Database (gnomAD, http://gnomad.broadinstitute.org/), and those that are thought to have no functional impact (e.g. synonymous mutations, non-coding mutations) were excluded. Sequence variants were further screened based on pathogenicity prediction using online tools SIFT (http://sift.jcvi.org), Polyphen2 (http://genetics.bwh.harvard.edu/pph2/), Mutation Taster (https://www.mutationtaster.org/), and Combined Annotation Dependent Depletion (CADD, https://cadd.gs.washington.edu), clinical phenotypes, inheritance and literature reports. Validation of candidate variants was performed by Sanger sequencing.

## Results

### Clinical presentation

**Fig. 1:**
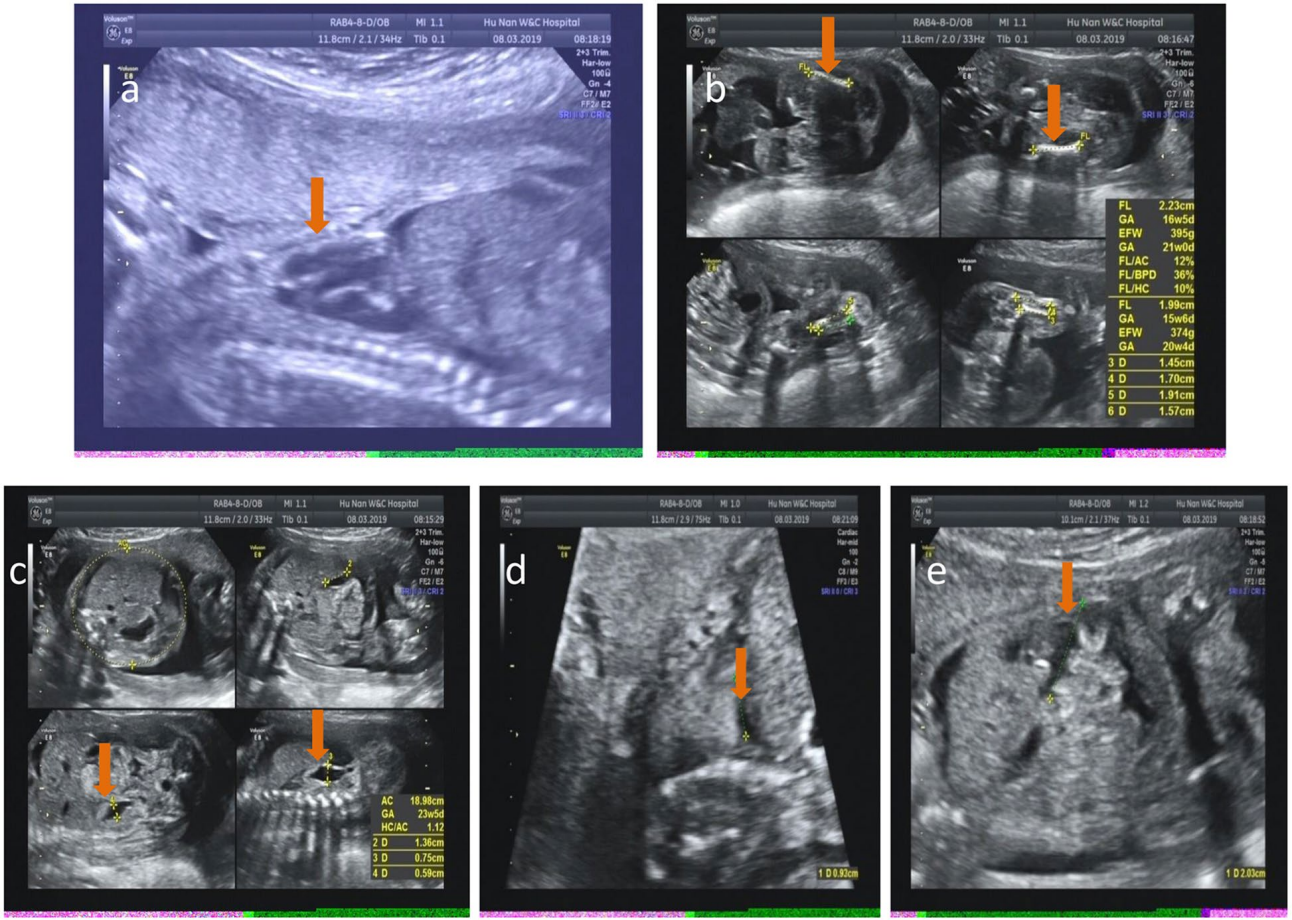
Ultrasound findings of the fetus in patient 1. Narrow thorax (**a**), shortened femur (**b**), bilateral pyelectasis (**c**), pleural effusion (**d**), and peritoneal effusion (**e**) are indicated by red arrows

**Fig. 2:**
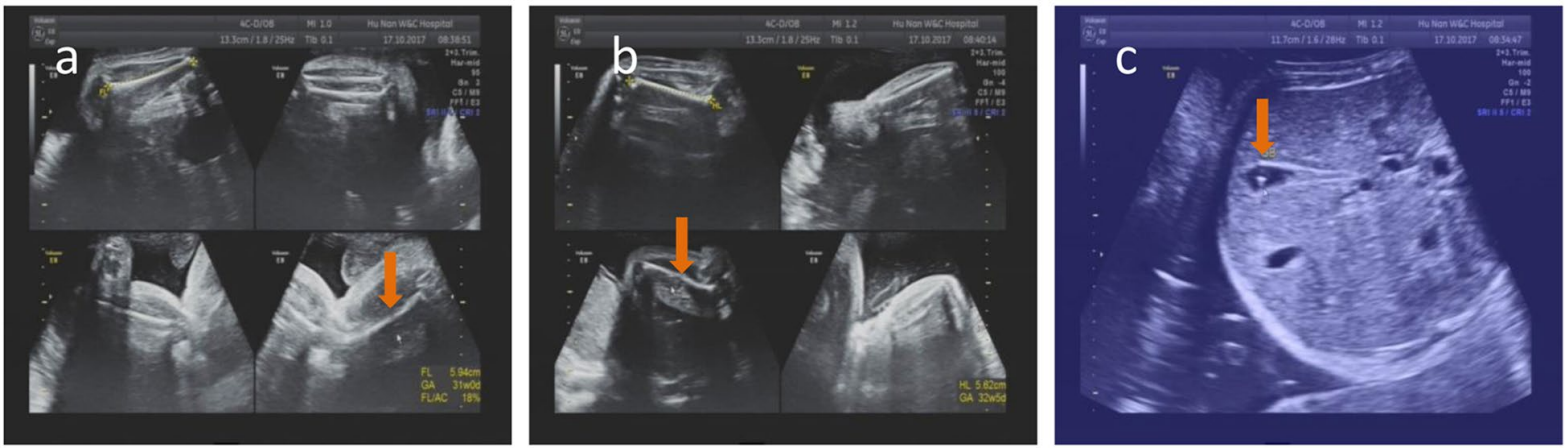
Ultrasound features of the fetus in patient 2. Short femurs (**a**) and humeri (**b**) and high echo spot in gallbladder crystals (**c**) are indicated with red arrows

**Fig. 3:**
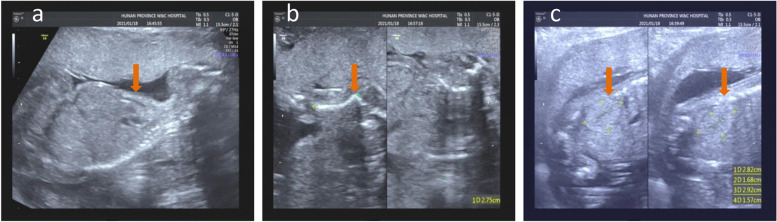
Ultrasound features of the fetus in patient 3. Narrow thorax (**a**), short bell-shaped ribs (**b**), and enlarged kidneys and enhanced renal parenchymal echo (**c**) are indicated with red arrows

**Fig. 4:**
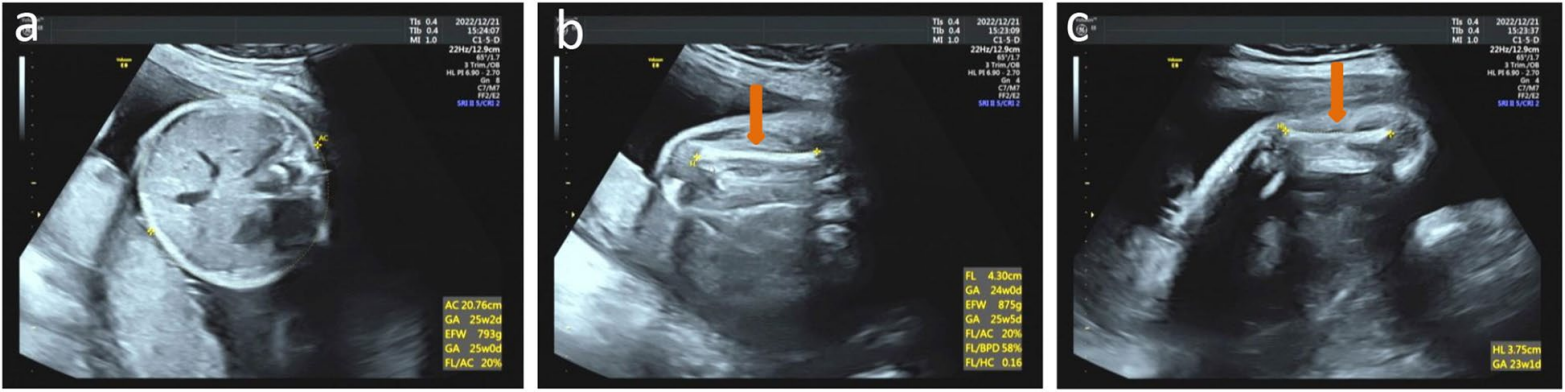
Ultrasound features of the fetus in patient 4. Narrow abdominal circumference is revealed (**a**). Short femurs (**b**) and humeri (**c**) are indicated with red arrows

The clinical features of the prenatal cases were given in Table 1 and Figs. 1, 2, 3 and 4, The major and common ultrasound anomalies for the four unrelated fetuses included short long bones of the limbs and narrow thorax.

**Table1:** clinical features and genetic findings of the prenatal cases with skeletal dysplasia

Case	Maternal age & history	Gestation	Ultrasound abnormalities	Pregnancy outcome	Variant	Fullfilled ACMG rules	Classification
1	26 yrs, gravid 1 para 0	25 weeks	A narrow thorax and short limbs (humeri lengths 2.0 cm and femurs lengths 2.2 cm, < -3.18 SD) with pleural and peritoneal effusion and bilateral pyelectasis; no polydactyly and other remarkable malformations	Terminated at 28 weeks	***DYNC2H1* NM_001080463.2**		
					c.3133C > T;p.Gln1045Ter	The variant causes a C-terminally truncated protein at codon 1045. The full-length DYNC2H1 protein consists of 4307 amino acid residues, and thus the variant is considered loss-of-function. (PVS1) The variant is absent in population databases including ExAC, 1000G, and gnomAD. (PM2)	Likely Pathogenic
					c.6809G > A;p.Arg2270Gln	The variant is absent in population databases including ExAC, 1000G, and gnomAD. (PM2) The online tool Mutation Taster predicts that the arginine is conserved across species and glutamine substitution is disease causing. (PP3)	Uncertain significance
2	29 yrs, gravid 1 para 0	38 weeks + 5 days	Short long bones of the limbs (humeri lengths 5.6 cm and femurs lengths 6.1 cm, < -3.13 SD), high echo spot in gallbladder crystals, and high density of umbilical coiling. Amniotic fluid index was 22.1 cm	Naturally delivered at term when the diagnostic ES was still undergoing	***IFT172* NM_015662.3**		
					c.1513C > T;p.Arg505Trp	The variant possesses a frequency of 0.0003 and 0.0009, respectively, in ExAC and gnomAD.(PM2)	Uncertain significance
					c.4540-5 T > A	The variant has not been reported in ExAC, 1000G and gnomAD. (PM2) The CADD online tool analysis suggests that the -5 position is a splice acceptor site, mutation of which probably affects RNA splicing. (PP3)	Uncertain significance
3	24 yrs, gravid 1 para 0	22 weeks + 6 days	Shortened ribs (bell-shaped), narrow thorax, shortened long bones of the limbs (humeri lengths 2.8 cm and femurs lengths 3.2 cm, < -3.04 SD), enlarged kidneys, and enhanced renal parenchymal echo	Terminated at 25 getation weeks before the molecular diagnosis was completed	***WDR19* NM_025132.4**		
					c.2363 + 1G > A	The variant destroys the canonical splice donor site in intron 20 and theoretically results in absent or disrupted protein product. (PVS1) The variant has a frequency of 4.76e-05 in gnomAD. (PM2)	Likely pathogenic
					c.2596G > C;p.Gly866Arg	The variant has not been reported in the ExAC, 1000G and gnomAD databases. (PM2) In silico analysis (Mutation Taster) predicts that the substitution is deleterious to the protein. (PP3)	Uncertain significance
4	28 yrs	28 weeks after ovulation induction	Intrauterine growth restriction (IUGR), abdominal circumference < 3^rd^ percentile and femurs lengths < -3.77 SD	Loss of follow-up	***DYNC2H1* NM_001080463.2**		
					c.1390C > T;p.Arg464Ter	The variant produces an early truncated protein with a loss of the majority of amino acid residues, and thus the variant is considered non-functional. (PVS1) The variant has been reported in gnomAD with a frequency of 5.59e-05, but not in ExAC or 1000G. (PM2)	Likely pathogenic
					c.337C > T;p.Arg113Trp	The variant is listed in ExAC and gnomAD with a frequency of 1.66e-05 and 4.80e-05,respectively.(PM2) The variant has previously been reported to be in trans position with the pathogenic variant c.3353del (p.Ser1118IlefsTer46) to cause ATD (PM3)	Uncertain significance

### Genetic findings

Karyotypes were normal, and no pathogenic CNVs were detected by SNP array in the four unrelated fetuses. The top 10 candidate variants for individual fetuses as revealed by ES are listed in Supplementary Material Table S2.

**Fig. 5:**
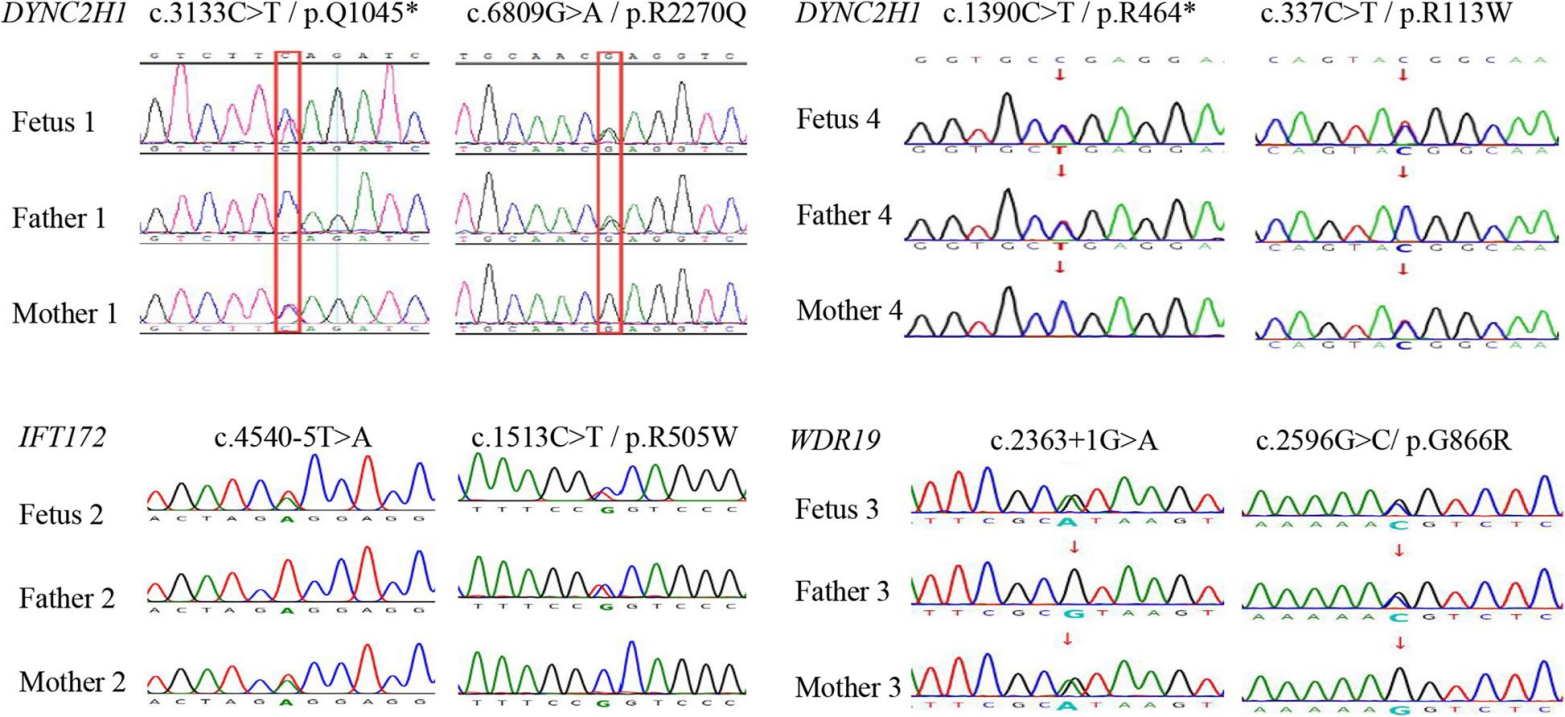
Sanger sequencing confirmed the candidate causative mutations in individual fetuses

The fetus 1 carried a heterozygous *DYNC2H1* variant NM_001080463.2:c.3133C > T p.(Gln1045Ter) in one allele and a heterozygous *DYNC2H1* variant NM_001080463.2:c.6809G > A p.(Arg2270Gln) in the second allele, which are maternally and paternally inherited, respectively (Fig. 5). The two variants were not found in public databases HGMD professional and Varsome (accessed on Nov 11^th^, 2023). According to the ACMG guidelines, the c.3133C > T variant was regarded as likely pathogenic, whereas the c.6809G > A variant was of uncertain significance (Table 1). The identified genotype and ultrasonic features of the fetus 1 support a diagnosis of SRTD3.

The fetus 2 was biallelic heterozygous for *IFT172* NM_015662.2:c.4540-5 T > A and NM_015662.2:c.1513C > T p.(Arg505Trp) (Fig. 5). The c.4540-5 T > A variant had not been indexed by HGMD professional and Varsome, whereas the c.1513C > T variant was previously reported by our team [12]. Based on the ACMG guidelines, the variants c.4540-5 T > A and c.1513C > T are considered as VUS (Table 1). The identified genotype and ultrasonic features of the fetus 2 support a diagnosis of SRTD10.

The heterozygous variants *WDR19* NM_025132.4:c.2596G > C p.(Gly866Arg) and NM_025132.4:c.2363 + 1G > A, which are of maternal and paternal origins, respectively, were identified in the fetus 3 (Fig. 5). The c.2596G > C variant was not documented in the databases HGMD professional and Varsome. The c.2363 + 1G > A variant is seen recurrently in publications [9, 13–16]. In terms of the ACMG guidelines, the c.2363 + 1G > A variant is considered as likely pathogenic (PVS1 + PM2_-_p), the c.2596G > C variant is of uncertain significance (VUS) (Table 1). Notably, the c.2363 + 1G > A variant is classified as pathogenic/likely pathogenic in the ClinVar database (https://www.ncbi.nlm.nih.gov/clinvar/, Variation ID: 280,765). The c.2597G > A variant of *WDR19*, with identical amino acid change (p.Gly866Arg) to the c.2596G > C variant, has been documented in ClinVar (Variation ID: 1,494,663) and classified as VUS. Mutations in *WDR19* are associated with autosomal recessive cranioectodermal dysplasia 4 (MIM 614378), SRTD5 (MIM 4376), nephronophthisis 13 (MIM 614377) and Senior-Loken syndrome (MIM 616307). The genotype and phenotype of the fetus 3 suggests a diagnosis of SRTD5.

Heterozygous variants *DYNC2H1* NM_001080463.2:c.1390C > T p.(Arg464Ter) and NM_001080463.2:c.337C > T p.(Arg113Trp) were identified in the fetus 4 (Fig. 5). The c.1390C > T variant was not found in public databases HGMD professional and Varsome, and the c.337C > T variant was previously reported [9]. Based on the ACMG standards, the c.1390C > T variant is classified as likely pathogenic (PVS1 + PM2_-_p). It has been previously reported that the c.337 C > T variant is in trans position to the c.3353del (p.Ser1118IlefsTer46) variant in an individual diagnosed with ATD [9]. Therefore, the c.337C > T variant is classified as VUS (Table 1). In addition, the c.337C > T variant is graded as disease-causing mutation (DM) in HGMD. The genotype and phenotype of the fetus 4 supports a diagnosis of SRTD3.

## Discussion

In the present study, differential diagnosis of ultrasound-indicated fetal skeletal dysplasias in four unrelated fetuses was achieved by using ES, suggesting that ES is an efficient and cost-effective method for prenatal diagnosis of rare genetic skeletal disorders.

Compound heterozygous (or homozygous) mutations in *DYNC2H1* or digenic biallelic mutations in *DYNC2H1* and *NEK1* have previously been identified as a genetic cause for SRPS type III, the most common type of SRPS [17, 18]*. DYNC2H1* encodes cytoplasmic dynein 2 heavy chain 1, which acts as a motor for intraflagellar retrograde transport and take participation in cilia biogenesis [19]. In a previous study to screen causal genes for ATD, EVC and SRPS spectrum in 152 unrelated families by using exome sequencing, mutations in *DYNC2H1* were found in 43 SRPS families (40 with SRPS type III), and 110 different pathogenic mutations in *DYNC2H1* were identified, two thirds of which were missense mutations and mainly clustered in the N-terminal tail, the AAA2-4 ATPase domains and the conserved C-terminal domain (C domain) [9]. In the present study, the p.Arg2270Gln, p. Gln1045Ter and p.Arg464Ter mutations are located in the AAA3 domain, the interval region between the DHC_N1 and DHC_N2 domains and the DHC_N1 domain, respectively. Interestingly, the biallelic heterozygous mutations in *DYNC2H1* in the two unrelated fetuses both comprise a loss-of-function (nonsense) mutation and a missense mutation, in line with a previous report that biallelic loss of function of DYNCH2H1 might be embryo lethal [9].

The *IFT172* gene has been associated with autosomal recessive Bardet-Biedl syndrome [20], non-syndromic retinitis pigmentosa [21] and SRTD10 [22]. In a previous study, homozygous or compound heterozygous mutations in *IFT172* were detected in 12 families with affected individuals diagnosed with asphyxiating thoracic dystrophy (ATD; also known as Jeune syndrome) or Mainzer-Saldino syndrome (MZSDS). The patients were characterized by abnormalities of the thorax and/or long bones with involvement of other organs such as kidney, liver, or retina. In the present study, no remarkable visceral anomalies were observed in the fetus 2.

The WD domain repeat 19 (*WDR19*) gene, also known as *IFT144*, belongs to the WD (tryptophan-aspartic acid) repeat family. Mutations in *WDR19* have been associated with a broad spectrum of ciliopathies, such as Sensenbrenner syndrome (cranioectodermal dysplasia), Jeune syndrome, Senior-Loken syndrome (nephronophthisis and pigmentary retinopathy), and nonsyndromic asthenoteratospermia [13, 23, 24]. In our study, renal involvement was observed in the fetus 3. This finding is in line with the fact that kidney disease is frequently seen in WDR19-related ciliopathies.

At prenatal stage, the information regarding the clinical phenotype of fetus might be incomplete due to later stage-specific onset of certain anomalies or limitations from ultrasound examination. Genetic analysis would contribute to understand the etiology and predict prognosis of the affected fetus, as well as guide the next pregnancy. Moreover, proper genetic counseling for the affected family is essential in the case of rare genetic diseases, since parental genetic screening/diagnosis is the best strategy for managing these diseases currently having no therapy [25–27]. Reporting additional cases on fetal skeletal ciliopathies and causal genes would help identify the genotype–phenotype correlations and lead to clinical trials in the future [28].

Collectively, our case series provide information regarding the novel variants of ciliopathies-associated genes *DYNC2H1*, *IFT172*, and *WDR19*, and emphasize the importance of both clinical and genetic findings in the setting of prenatal diagnosis for skeletal disorders.

### Supplementary Information

**Additional file 1: Supplementary Table S1. **The phenotype-genotype relationships of short-rib thoracic dysplasia (SRTD) with or without polydactyly types 1-23.

**Additional file 2: Supplementary Table S2. **Top 10 candidate mutations as revealed by WES.

## Data Availability

The data that support the findings of this study are available via the BioProject database (Accession: PRJNA878618) at https://ncbi.nlm.nih.gov/bioproject/.
