# Supplementary material for: Clinical features and genetic analysis of a case series of skeletal ciliopathies in a prenatal setting
Source: BMC Med Genomics. 2023 Dec 7;16:318. doi: 10.1186/s12920-023-01753-y (PMC10704717; doi:10.1186/s12920-023-01753-y)
Supplement: Supplementary file 1 — Additional file 1: Supplementary Table S1. The phenotype-genotype relationships of short-rib thoracic dysplasia (SRTD) with or without polydactyly types 1-23. [file 12920_2023_1753_MOESM1_ESM.docx]

**Supplementary Table S1** The phenotype-genotype relationships of short-rib thoracic dysplasia (SRTD) with or without polydactyly types 1-23

| Phenotype | OMIM # | Gene | OMIM* |
| --- | --- | --- | --- |
| SRTD1 | 208500 | gene locus: 15q13 |  |
| SRTD2 | 611263 | *IFT80* | 611177 |
| SRTD3 | 613091 | *DYNC2H1* | 603297 |
| SRTD4 | 613819 | *TTC21B* | 612014 |
| SRTD5 | 614376 | *WDR19* | 608151 |
| SRTD6 | 263520 | *NEK1* | 604588 |
| SRTD7 | 614091 | *WDR35* | 613602 |
| SRTD8 | 615503 | *WDR60* | 615462 |
| SRTD9 | 266920 | *IFT140* | 614620 |
| SRTD10 | 615630 | *IFT172* | 607386 |
| SRTD11 | 615633 | *WDR34* | 613363 |
| SRTD12 | 269860 | *SRTD12* | 269860 |
| SRTD13 | 616300 | *CEP120* | 613446 |
| SRTD14 | 616546 | *KIAA0586* | 610178 |
| SRTD15 | 617088 | *DYNC2LI1* | 617083 |
| SRTD16 | 617102 | *IFT52* | 617094 |
| SRTD17 | 617405 | *TCTEX1D2* | 617353 |
| SRTD18 | 617866 | *IFT43* | 614068 |
| SRTD19 | 617895 | *IFT81* | 605489 |
| SRTD20 | 617925 | *INTU* | 610621 |
| SRTD21 | 619479 | *KIAA0753* | 617112 |
| SRTD22 | 225500 | *EVC2* | 607261 |
| SRTD23 | 225500 | *EVC* | 604831 |
